# Supplementary material for: Air-liquid interface model for influenza aerosol exposure in vitro
Source: J Virol. 2025 Jun 3;99(7):e00619-25. doi: 10.1128/jvi.00619-25 (PMC12282172; doi:10.1128/jvi.00619-25)
Supplement: Fig. S1 — Formulas and calculations. [file jvi.00619-25-s0001.pdf]

**Supplementary Figure 1. Calculations and exposure chamber parameters to determine the cumulative deposition of virus aerosols per 6 well plate.**

(1) $\text{Emissions}\left(\frac{\text{TCID50}}{\text{s}}\right) = \frac{\text{vol nebulized (mL)} \times \text{initial titer}\left(\frac{\text{TCID50}}{\text{mL}}\right)}{\text{time nebulizer (min)} \times 60\ (\text{s})}$

$TCID_{50}$ = virus infectivity in units of 50% tissue culture infectious dose
vol = volume of liquid nebulized
time nebulizer = duration that the nebulizer ran.

(2) $\text{Vcham}\ (\text{m}^3) = \text{L (m)} \times \text{W (m)} \times \text{H (m)}$

Vcham = volume of chamber
L, W, H = length, width, and height of chamber

(3) $\text{Vd dry aerosol}\left(\frac{\text{m}}{\text{s}}\right) = \frac{(\text{size of dry aerosol (um)} \times 0.000001)^2 \times 9.81 \times 1000 \times \text{C}}{(18 \times 0.0000185)}$

Vd dry aerosol = settling velocity of dry aerosol particles
C = Cunningham slip correction factor

(4)

$\text{Virus}\left(\frac{\text{TCID50}}{\text{m}^3}\right) = \frac{\text{Emissions}\left(\frac{\text{TCID50}}{\text{s}}\right)}{\text{Vd dry aerosol}\left(\frac{\text{m}}{\text{s}}\right) \times \text{L (m)} \times \text{W (m)} + \text{Qsamp}\left(\frac{\text{m3}}{\text{s}}\right)} \times \left(1 - e^{\frac{-(\text{Vd dry aerosol}\left(\frac{\text{m}}{\text{s}}\right) \times \text{L cham (m)} \times \text{W (m)} + \text{Qsamp}\left(\frac{\text{m3}}{\text{s}}\right)}{\text{Vcham}(\text{m}^3)\ \text{x t(s)}}}\right)$

Virus = concentration of infectious virus in the air
Qsamp = volume flow rate of aerosol sampler

(5) $\text{flux Virus}\ \frac{\text{TCID50}}{\text{m2 x s}} = \text{Vd dry aerosol}\left(\frac{\text{m}}{\text{s}}\right) \text{x Virus}\left(\frac{\text{TCID50}}{\text{m}^3}\right)$

flux Virus = deposition rate of infectious virus per unit area per unit time

(6) $\frac{\text{deposition}}{\text{6 well}}\left(\frac{\text{TCID50}}{\text{min}}\right) = \text{flux Virus}\ \frac{\text{TCID50}}{\text{m2 x s}}\ \text{x}\ \text{Area}_{\text{well}}\ \text{x 60s}$

deposition/6 well = deposition rate of infectious virus on a 6-well plate
$\text{Area}_{\text{well}}$ = area of the wells in a 6-well plate

(7) $\text{Cumulative}\frac{\text{deposition}}{\text{6 well}}\ (\text{TCID50}) = \sum_0^{15} \frac{\text{deposition}}{\text{6 well}}\left(\frac{\text{TCID50}}{\text{min}}\right)$

Cumulative deposition/6 well = cumulative deposition of infectious virus on a 6-well plate over the experimental period of 15 minutes.

**Parameters:**
(Eq 1) Volume nebulized (vol nebulized) = 6 mL
(Eq 1) Time nebulizer = 15 min
(Eq 2) L = 0.487 m
(Eq 2) W = 0.343 m
(Eq 2) H = 0.355 m
(Eq 3) Size of dry aerosol = 1.5 µm, mode of size distribution determined by measurement using the Aerotrak
(Eq 4) Qsamp = 16 L/min = 0.000267 $m^3/s$
(Eq 4) time nebulizer (t(s)) = increases every 60s until 15min total time
(Eq 5) $\text{Area}_{\text{well}}$ = 0.001 $m^2$ (6-well plate) or 0.0001 $m^2$ (Transwell)
